# Evidence from a rare case study for Hebbian-like changes in structural connectivity induced by long-term deep brain stimulation

**DOI:** 10.3389/fnbeh.2015.00167

**Published:** 2015-06-30

**Authors:** Tim J. van Hartevelt, Joana Cabral, Arne Møller, James J. FitzGerald, Alexander L. Green, Tipu Z. Aziz, Gustavo Deco, Morten L. Kringelbach

**Affiliations:** ^1^Department of Psychiatry, University of OxfordOxford, UK; ^2^Center of Functionally Integrative Neuroscience (CFIN), Aarhus UniversityAarhus, Denmark; ^3^Center of Brain and Cognition, Theoretical and Computational Neuroscience Group, Universitat Pompeu FabraBarcelona, Spain; ^4^Nuffield Department of Surgical Sciences, John Radcliffe HospitalOxford, UK; ^5^Institució Catalana de la Recerca i Estudis Avançats (ICREA), Universitat Pompeu FabraBarcelona, Spain

**Keywords:** deep brain stimulation, Hebbian-like learning, Parkinson’s disease, DTI, subthalamic nucleus

## Abstract

It is unclear whether Hebbian-like learning occurs at the level of long-range white matter connections in humans, i.e., where measurable changes in structural connectivity (SC) are correlated with changes in functional connectivity. However, the behavioral changes observed after deep brain stimulation (DBS) suggest the existence of such Hebbian-like mechanisms occurring at the structural level with functional consequences. In this rare case study, we obtained the full network of white matter connections of one patient with Parkinson’s disease (PD) before and after long-term DBS and combined it with a computational model of ongoing activity to investigate the effects of DBS-induced long-term structural changes. The results show that the long-term effects of DBS on resting-state functional connectivity is best obtained in the computational model by changing the structural weights from the subthalamic nucleus (STN) to the putamen and the thalamus in a Hebbian-like manner. Moreover, long-term DBS also significantly changed the SC towards normality in terms of model-based measures of segregation and integration of information processing, two key concepts of brain organization. This novel approach using computational models to model the effects of Hebbian-like changes in SC allowed us to causally identify the possible underlying neural mechanisms of long-term DBS using rare case study data. In time, this could help predict the efficacy of individual DBS targeting and identify novel DBS targets.

## Introduction

Deep brain stimulation (DBS) is a well-established treatment for several neurological conditions including Parkinson’s disease (PD; Benabid et al., 2009; Lozano and Lipsman, 2013). However, the underlying neural mechanisms of DBS and its long-term effects on brain connectivity remain unclear. This limitation restricts the efficacy of DBS since the identification of individual DBS targets and the settings of stimulation parameters cannot be optimally performed beforehand. Uncovering these aspects will improve the clinical benefits of DBS in the treatment of such diseases.

In general terms, the effects of DBS must be closely linked to at least three factors: (1) the stimulation parameters such as frequency, amplitude, pulse width and duration; (2) the physiological properties of the neural tissue (which may be dependent on disease state); and (3) the interactions between the electrode and the surrounding neural tissue and specific anatomy of the targeted region (Kringelbach et al., 2007, 2010). This third factor includes the extended brain-wide connectivity pattern from the electrode where this specific structural “fingerprint” of connectivity is an important factor for the efficacy (Fernandes et al., 2015). Thus rather than solely acting locally, the evidence clearly shows that DBS affects a network of neural elements; foremost myelinated axons,and to a lesser degree cell bodies. Thus the most likely mechanism of DBS is through stimulation-induced modulation of the activity of macroscopic brain networks (Montgomery and Baker, 2000; Vitek, 2002; McIntyre and Hahn, 2010; Kringelbach et al., 2011). This has been confirmed by optogenetic experiments in rodents which show that the therapeutic effects within the subthalamic nucleus (STN) can be accounted for by direct selective stimulation of afferent axons projecting to this region (Gradinaru et al., 2009). It is not clear, however, which of the many connections from a given DBS target are most influential in providing a clinical benefit and whether DBS creates long-term changes in brain connectivity.

In this case study, we exploit the unique opportunity of having preoperative and five-month postoperative diffusion tensor imaging (DTI) data from a single patient with DBS in the STN for the treatment of PD. This allowed us to reconstruct the three-dimensional networks of white-matter connections—or structural connectivity (SC)—before and after long-term DBS, which we used to simulate the corresponding spontaneous dynamics using a whole-brain computational model (Deco et al., 2013b).

We hypothesized that computational modeling of the effects of changes in SC following DBS would allow us to identify the significant, causal neural mechanisms of DBS compared to not applying DBS. In order to do this, we calculated the change of brain activity induced by the DBS by simulating resting state activity before and after DBS. We analyzed under which conditions the predicted change of neuronal activity correlates with the changes in the SC observed 5 months after operation, i.e., assuming the existence of Hebbian-like learning, where the weights in the model were changed in a Hebbian-like manner. We were able to test this by systematically changing the weight of the structural connections from the STN to itself and its known projections in the putamen, caudate and thalamus. We thus used established principles of Hebbian-like learning to change the functional dynamics between STN and its projections to find the optimal weights that best describe the long-term changes in SC induced by DBS.

Finally, we calculated the overall impact of long-term DBS on measures of integration and segregation, representing two fundamental aspects of brain organization and information processing (Deco et al., 2015).

## Materials and Methods

### Brief Description of Analysis Pipeline

**Figure 1:**
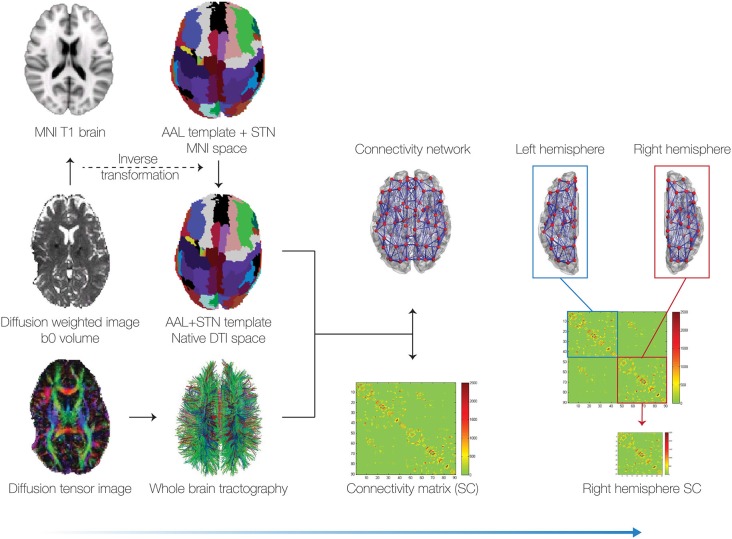
**Pipeline for generating the connectivity matrices**. We collected pre and post diffusion tensor imaging (DTI) data from a patient and then generated the whole-brain tractography for all voxels (bottom left). In addition to the 90 cortical and subcortical regions in the automated anatomical labeling (AAL) template, we incorporated the subthalamic nucleus (STN), based on the binarised version of a probabilistic mask (Forstmann et al., 2012). This resulted in a total of 92 cortical and subcortical regions (46 per hemisphere). We reconstructed the whole-brain connectivity matrix between these 92 brain regions using the methods described in van Hartevelt et al. (2014). Due to the electrode artifact in the left hemisphere (see bottom left), in the subsequent analyses we only used the right hemisphere connectivity matrices.

**Figure 2:**
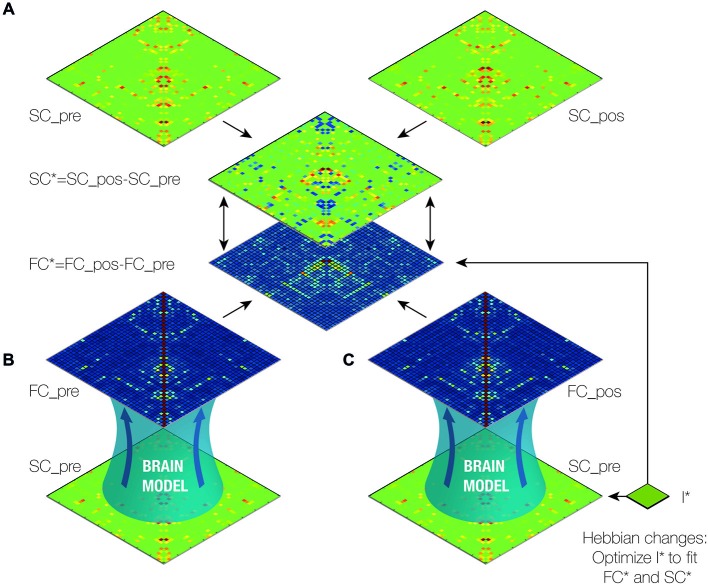
**Outline of brain model analysis and optimization**. **(A)** First, we created the difference (SC*) between the pre- and post-operative structural connectivity (SC) matrices (SC_pos-SC_pre; see Figure 1) (van Hartevelt et al., 2014). **(B)** The functional connectivity matrix (FC_pre) was generated with a dynamic mean-field (DMF) model using SC_pre. **(C)** We then iteratively generated the functional connectivity post-DBS (FC_pos) using the computational model with SC_pre and the I*, the weights of the known connections from the STN. We subsequently optimized I* such that the difference between FC_pre and FC_pos (FC*) was made to fit SC*. In this way, we estimated the optimal working weights from the STN. Finally, we calculated the contribution of each of the connections from the STN to the changes in functional connectivity caused by DBS by varying the weights of each of the connections and measuring the impact on the fit (see Figure 3).

**Figure 3:**
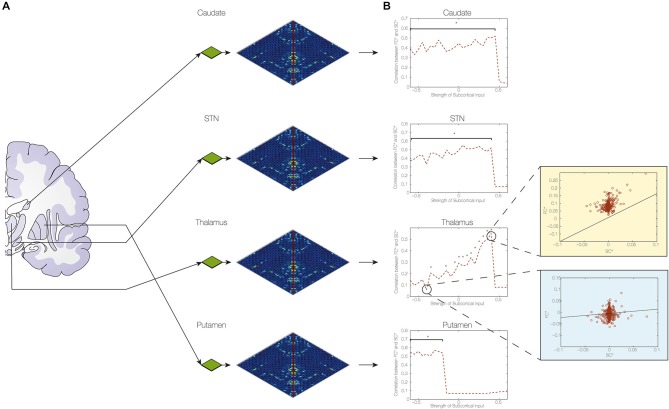
**Determining the contribution of subcortical connections of STN**. **(A)** We found the optimum fit of I* and then varied each of the model parameters of the connections from the STN to the putamen, caudate, thalamus and STN itself to estimate their contributions to the changes in functional connectivity caused by DBS. Compared to no DBS, the optimum fit was highly significant (*p* < 10^–7^). **(B)** The figure clearly demonstrates the importance of the changes in connectivity weights to the thalamus and the putamen with the consequences of the best fit in thalamic connectivity shown in top right (light yellow) and poorest fit shown in bottom right (light blue) (with a star indicating significance levels of *p* < 0.05).

In this case study, we investigated the underlying neural mechanisms of long-term DBS in terms of connectivity changes by using a computational dynamic mean field (DMF) model (Deco and Jirsa, 2012; Deco et al., 2013b) on pre- and post-operative DTI data. Briefly, we analyzed the data as follows (and further described in details below):

1. *Structural connectivity*. Structural connectomes were constructed for the pre- and post-operative data. We also computed the difference between pre- to post-operative connectomes (SC*). In addition, we generated a group-averaged connectome from healthy participants to optimize the DMF model (see Figures 1, 2A and “Structural Brain Networks” Section).
2. *Computational dynamical mean field model*. The DMF model was optimized in the same way as described in our previous case-study (van Hartevelt et al., 2014), i.e., by fitting the FC and SC from healthy individuals [see Figure 2B and “Model of Spontaneous Functional Connectivity (FC)” Section]. As shown in previous studies (Cabral et al., 2014), this model accurately captures many features of the resting-state dynamics of FC.
3. *Finding optimal fit of model*. The DMF model was applied to the pre-operative structural connectome (SC_pre) to generate the pre-operative functional resting-state activity (FC_pre). Crucially, we then added the four established targets of the DBS electrode in STN (itself, thalamus, caudate and putamen) (I*), and used the DMF model on FC_pre with I* to generate the putative post-operative functional resting-state activity (FC_pos). This allowed us to compute the difference between the pre- and post-operative FC matrices (FC*). We then systematically optimized the parameters of I* to find the best possible fit, i.e., we compared the effects of changing I* to get the maximal correlation between each of the SC* and FC* pairs (see Figure 2C).
4. *Determine the causal contribution of each STN projection*. Once we had found the optimal fit of weights for STN projections (I*), we systematically varied each of the four structural weights while holding the other weights constant (see Figure 3A). This allowed us to determine which of these projections best predict the effects of STN DBS on postoperative FC.
5. *Determine the global influence of long-term Hebbian-like changes in SC following DBS*. We used the DMF model to compute measures of integration and segregation of information processing in the pre- and post-operative brain compared to the healthy brain (see “Functional Measures” Section).

### Patient and Healthy Participants

We acquired DTI data preoperatively and 5 months postoperatively after continuous DBS in a 45-year-old female PD patient. The patient’s main symptoms included on/off fluctuations and dyskinesias. The patient received continuous DBS *on* and *off* over 5 months during stimulation optimization. In order to plan a lead revision (warranted by adverse side effects including emotional lability) post DBS DTI data were acquired. The new target for the lead revisions was the internal Globus Pallidus. Following DBS surgery, the medication was continued with Pramipexole 0.7 one and a half tablets thrice daily. Stalevo was reduced to 50 mg thrice daily (from 150 mg in the morning, 50 mg twice daily and 100 mg in the evening) and Amantadine was stopped completely (from 100 mg twice daily). During the periods when the DBS was turned *off*, the patient was advised to return to her preoperative medication regime. Whereas right DBS lead titration resulted in symptom reduction, left DBS lead titration was more problematic and resulted in adverse side effects. A possible explanation for this is the suboptimal positioning of the electrode. During the 5 months between DTI acquisitions the stimulation parameters changed due to fine tuning and titration of the DBS electrodes. Additional DTI data were acquired for nine healthy participants (three females, age range 22–40 years). This study was approved by the National Research Ethics Service (NRES) committee South Central—Berkshire in Bristol.

### Surgical Procedure

The DBS electrodes were implanted in the bilateral STN. Prior to surgery, anatomical T1 and T2 MRI scans with 1 × 1 × 1 mm voxel size were acquired for electrode implant protocol planning. The surgery was performed under local anesthesia using a Cosman–Roberts–Wells stereotactic. See Kringelbach et al. (2009) for more details on the surgical procedure.

### Image Acquisition

All DTI data for the patient and healthy participants were acquired on a Philips Achieva 1.5 Tesla MR scanner in Oxford. Whole brain diffusion weighted imaging was performed using a single-shot echo planar sequence. The scanning parameters were echo *time* (TE) = 65 ms, repetition *time* (TR) = 9390 ms, reconstructed matrix 176 × 176 and reconstructed voxel size of 1.8 × 1.8 mm and slice thickness of 2 mm. We used 33 optimal nonlinear diffusion gradient directions (*b* = 1200 s/mm^2^) and one non-diffusion weighted volume (*b* = 0) for the DTI acquisition. The post-DBS DTI data was acquired with DBS turned off.

### Structural Brain Networks

The whole-brain structural networks were constructed in a two-step process used successfully in previous published studies (Cabral et al., 2013; van Hartevelt et al., 2014; Fernandes et al., 2015). First, the brain was parcellated into different regions or nodes. Secondly, the edges, or connections between nodes, were estimated using probabilistic tractography (Figure 1). Each of these two steps is described in detail in the following.

#### Parcellation of the Brain

The brain was parcellated into 90 cortical and subcortical regions (45 for each hemisphere) using the automated anatomical labeling (AAL) template (Tzourio-Mazoyer et al., 2002). Additionally a mask of the STN from Forstmann et al. (2012) was incorporated to get a total of 92 cortical and subcortical regions (46 per hemisphere). In order to preserve as much information as possible, the parcellation of the brain was done in DTI native space.

We used the Flirt tool (FMRIB, Oxford; Jenkinson et al., 2002) for linearly coregistration of the b0 image in DTI space to the T1 structural image. The T1 image was in turn coregistered to the T1 template of ICBM152 in MNI space (Collins et al., 1994). The resulting transformations were concatenated and inversed and subsequently applied to transform the AAL template (Tzourio-Mazoyer et al., 2002) and the STN masks (Forstmann et al., 2012) from MNI space to DTI native space. This transformation of the template was conducted using a nearest-neighbor interpolation method to ensure that the discrete labeling values were preserved.

To minimize the effects of the DBS electrode artifact, a binary mask of the electrode lead in the post-DBS DTI data was created. This mask was subtracted from the brain masks and data of the pre-DBS DTI data as well as from the DTI data of the healthy controls.

#### Whole-brain Network Connectivity

We used FMRIB’s Diffusion Toolbox (FDT), which is a part of FSL (version 5.0, FMRIB, Oxford)^1^ to process the DTI data. The preprocessing involved coregistering the diffusion-weighted images to a reference volume using an affine transformation for the correction of head motion. This step also includes an eddy current correction. Next, the local probability distribution of fiber direction was estimated at each voxel using the default bedpostx parameters of FSL v5.0 (Behrens et al., 2003). Using the parameter estimation from bedpostx, the probtrackx algorithm (allowing for automatic estimation of two fiber directions within each voxel) was used, improving the tracking sensitivity of non-dominant fibers in the brain (Behrens et al., 2007).

We estimated the connectivity probability by applying probabilistic tractography using the default sampling settings of 5000 streamline fibers per voxel. The connectivity from a seed voxel *i* to another voxel *j* was defined by the proportion of fibers passing through voxel *i* that reach voxel *j* (Behrens et al., 2007). In a brain region, or node, consisting of *n* voxels, 5000*n fibers were sampled. The connectivity *C_ij_* from region *i* to region *j* is calculated as the number of sampled fibers connecting the voxels in region *i* to the voxels in region *j* divided by *n*, with *n* being the number of voxels in the seed region *i*.

The connectivity value for a given region to each of the 91 other regions was calculated. As probabilistic tractography depends on the seeding location, the connectivity from *i* to *j* is not necessarily equivalent to that from *j* to *i*. The connectivity values are highly correlated though across the brain for all participants (the least Pearson *r* = 0.70, *p* < 10–50). The symmetrical, undirectional connectivity *C_ij_* between regions *i* and *j* was calculated by averaging the two connectivity values. Therefore, we considered the SC between the two areas, where *C_ij_* = *C_ji_*. The connectivity values were calculated using in-house Perl scripts and were normalized for the regions’ volume with *n* voxels. The connectivity matrices were created for the preoperative condition (*SC_pre*), the postoperative condition (*SC_pos*) and one average connectivity matrix for the healthy subjects (*SC_normal*).

Due to the artifact of the DBS lead in the left hemisphere in the postoperative DTI data (see bottom left of Figure 1), only the sub-network corresponding to the right hemisphere was considered. In other words, we only used the 46 × 46 matrix corresponding to the right hemisphere (without inter-hemispheric connections) and not the full 92 × 92 connectivity matrix as shown in Figure 1. All further analyses and simulations were done using the right hemisphere only for preoperative, postoperative and healthy connectivity matrices.

### Model of Spontaneous Functional Connectivity

To illuminate the impact of DBS induces local structural changes on whole-brain activity, we used a DMF model to simulate the spontaneous dynamics of each brain area in the structural connectome (Deco and Jirsa, 2012; Deco et al., 2013b). The model is a reduction of the spiking network model, which includes the whole dynamics of excitatory and inhibitory populations of spiking neurons interconnected by AMPA, GABA and NMDA receptors and their respective equations (Wong and Wang, 2006). It describes the mean field activity of each brain area with a single one-dimensional equation and the level of excitation/inhibition of each node is balanced in order to maintain negligible short-range correlations and long-range functional correlations are introduced by excitatory inputs received from coupled brain regions according to the structural connectome. Thus the global dynamics of coupled brain areas can be simply and consistently described by the following set of coupled differential equations:

(1) $$\frac{dS_{i}(t)}{dt}=-\frac{S_{i}}{\tau_{S}}+(1-S_{i})\gamma H(x_{i})+\sigma \upsilon_{i}(t),$$

(2) $$H(x_{i})=\frac{ax_{i}-b}{1-\exp\left(-d(ax_{i}-b)\right)},$$

(3) $$x_{i}=wJ_{N}S_{i}+GJ_{N}\sum_{j}C_{ij}S_{j}+I_{0},$$

where *H(x_i_)* and *S_i_* denote the population rate and the average synaptic gating variable at the local cortical area *i* (from 1 to *N* = 46 areas in our case), respectively. *w* = 0.9 is the local excitatory recurrence and *C_ij_* corresponds to the coupling weight between the areas *i* and *j*. Note that *C_ij_* is estimated from the SC, i.e., in proportion to the number of connections (or connectivity value) detected between areas *i* and *j*, and therefore this parameter is changed between pre-DBS, post-DBS and control data. G is the global coupling weight that scales all couplings uniformly. Parameter values for the input–output function (2) are a =270 VnC, b = 108 Hz, and d =0.154 s. The kinetic parameters are γ = 0.641/1000 (the factor 1000 is for expressing everything in ms), and τ_s_ =100 ms. The synaptic couplings are *J_N_* = 0.2609 nA and the overall effective external input is *I*_0_ = 0.3 nA. In equation (1) *υ_i_* is uncorrelated standard Gaussian noise and the noise amplitude at each node is σ = 0.001 nA.

We used the Balloon-Windkessel hemodynamic model to transform the mean field activity into (simulated) BOLD signal, This model describes the transduction of neuronal activity into BOLD signal (Friston et al., 2003). The level of synaptic activity in each brain region represented by the synaptic variable *S_i_*, is used to compute the BOLD-signal estimation in that specific brain region as in (Deco and Jirsa, 2012; Cabral et al., 2013; van Hartevelt et al., 2014). The simulated BOLD signal was down-sampled at 2 s to have the same temporal resolution typically used in fMRI. The simulated FC between all brain areas is obtained by computing the temporal correlation matrix of the simulated BOLD signals.

The optimal fit of the model was calculated using an exhaustive search, i.e., we fitted all combinations of inputs (STN, thalamus, putamen and caudate) scanning them from values of −0.6 to 0.6 in steps of 0.05. From this search, we found the optimal working point of the model which is where the strengths of inputs from those subcortical areas yielded a maximal correlation between the differences in FC (FC*) and SC (SC*), i.e., where there is a maximal Hebbian-like correlation.

In order to determine the contribution of each individual connection on this fit, one of each of the four individual inputs from these subcortical areas were systematically changed while the other points were fixed at the optimal fit. Thus to determine the influence of the STN, for example, we systematically changed the values of the STN from −0.6 to 0.6, while keeping the values of the other three regions for the optimal fit. This process was then repeated for the putamen, caudate and thalamus, while keeping all other inputs fixed at the values of the optimal fit.

### Functional Measures

Two fundamental aspects of brain organization are the segregation of brain areas (that differ in terms of local functional specialization) and their global integration during perception and behavior (Tononi et al., 1994; Deco et al., 2015). As such, we investigated how these properties were affected before the surgery and to what extent DBS helped recover them towards normal values.

#### Segregation

To measure the segregation, we first estimate the mutual information between BOLD signals, which can be easily calculated—assuming Gaussianity of the mean BOLD responses—using the following equation:

(4) $$I(\chi_{1};\chi_{2};\ldots ;\chi_{\text{N}})=-\log\left(det(C)\right),$$

where *x_i_* refers to the BOLD signal of the *i* = 1, N nodes (brain areas) of the brain network and *C* is the correlation matrix between them, which corresponds to the FC.

The input segregation is then simply defined by

(5) $$S=1-\frac{I}{I_{\max}},$$

where *I*_max_ is an appropriate normalization specifying the maximal mutual information condition.

#### Integration

We measure the integration of FC based on a measure of the size of the largest connected component. More specifically, for a given absolute threshold θ between 0 and 1, the functional connectivity matrix FC can be binarized (using the criteria |FC_ij_| < θ; which determines if it will be given a value of 0 and 1) and the largest component extracted as a measure of integration. The largest component is the length (number of nodes) of the connected sub-graph of the undirected graph defined by the binarized matrix (which itself is considered as an adjacency matrix). This is the largest sub-graph in which any two vertices are connected to each other by paths, and which connects to no additional vertices in the super-graph. Finally, to get a measure that is independent of the threshold, this curve can be integrated in the range of the threshold between 0 and 1. This integration measure is normalized by the maximal number of connected brain areas (that is, all *N* areas) for each integration step and by the number of integration steps such that the maximal integration is normalized to one.

### Network Measures from Graph Theory

The analyses of the DTI data resulted in three 46 × 46 matrices, *SC_pre*, *SC_pos* and *SC_normal*, which can be analyzed as graphs. The brain networks were characterized using common measures from graph theory, namely the connection density, global efficiency, clustering coefficient, small-world index and hierarchy. We used the Brain Connectivity Toolbox to calculate all these global graph measures (Rubinov and Sporns, 2010; Sporns, 2011). These graph measures have been used and described in a previous study of brain connectivity in DBS (van Hartevelt et al., 2014).

These global graph measures were calculated for all three different anatomical brain networks, i.e., *SC_pre*, *SC_pos* and *SC_normal*. The average and standard deviation of the individual outcomes were then calculated and reported.

## Results

We investigated the consequences of having a DBS electrode implanted in the STN using the SC matrices, *SC_pre* and *SC_pos*, arising from the DTI of a PD patient before and 5 months after DBS surgery. Since the SC remains mostly unchanged over relatively short periods of time in healthy subjects (Cheng et al., 2012), we considered the difference between these matrices, SC*, as alterations induced by DBS in the patient’s structural connectome (see Figure 2A).

A DMF model was used to run simulations (see “Materials and Methods” Section) first using the SC *SC_pre*, from which we obtained the resulting *FC_pre* (see Figure 2B). We then iteratively optimized the weights of the known connections from the STN, namely to the putamen, the thalamus, the caudate and the STN itself, to estimate *FC_pos* such that the difference between *FC_pre* and *FC_pos*, FC*, optimally fitted SC* (see Figure 2C). This step is based on the assumption that the global network dynamics of a brain working at the critical instability border amplifies the underlying structure of anatomical connections (Deco et al., 2013a). In other words, at the optimal working point, the FC maximally reflects the underlying SC and hence, FC* maximally reflects SC*.

Once the optimum fit was found, we investigated the contribution of each of these brain regions to the changes in FC caused by DBS (see Figure 3). We found that the optimal connection strengths were −0.5 for the putamen, 0.4 for the thalamus, 0.4 for the caudate and 0.3 for the STN. Using this optimal fit with these connection strength values, show how individual variation of the coupling strength values influences the level of Hebbian-like learning induced (Figure 3), i.e., the correlation between the change in SC (SC, SC_pos-SC_pre) and the change predicted by the simulation according to the FC (functional connectivity, DBS—no DBS). The figure shows the correlation as a function of connection strength, indicating with a star those with a significance value of *p* < 0.05. Only the connection weights to the thalamus show a sensitive influence on the induction or not of the overall plasticity (i.e., of inducing high correlations between changes in SC and predicted changes in FC), while the other subcortical nuclei are inducing the same level of plasticity if the weights are within the right range (e.g., putamen below −0.2). Thus the thalamus would appear to be the most critical location for the DBS to induce optimum changes.

**Figure 4:**
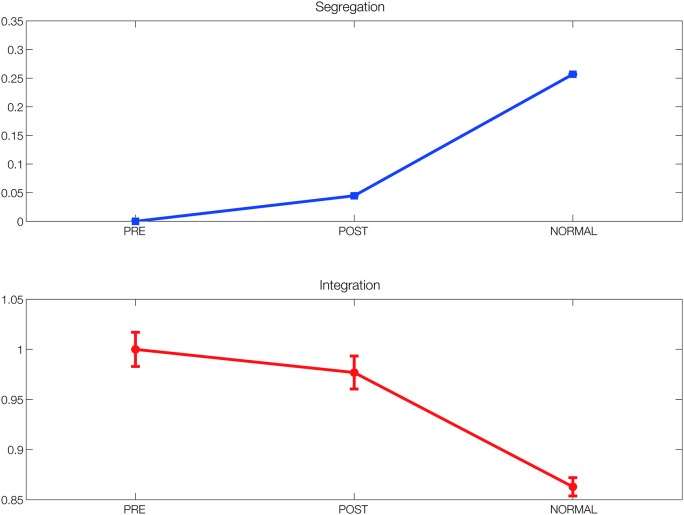
**Segregation and integration**. Segregation and integration measures in the simulated functional networks before and after DBS (FC_pre and FC_pos) compared to the ones found in the simulated functional network obtained using the connectivity from the healthy population (FC_normal). Both measures improved after 5 months of DBS, although not to the level found in the normal population.

To further investigate the structural changes induced by DBS in the functional networks in terms of segregation and integration of information processing, we estimated these two measures in FC_pre, FC_pos and FC_normal (the latter obtained with the model from SC_normal). As expected, we found that both the segregation and integration measures improved after 5 months of DBS, although not to the level found in the normal population (see Figure 4).

Finally, we used measures from graph theory to investigate the effects of long-term DBS on global network SC. The results that are reported in Table 1 show that there was no effect of DBS on global measures of connection density, average clustering coefficient, global efficiency, small-world index or hierarchy.

**Table 1:** **Graph theoretical measures for pre, post and normal connectomes**.

	SC_pre	SC_pos	SC_normal
Connection density (%)	45.09	45.94	46.51 (SD 8.35)
Average clustering coefficient	0.6680	0.6955	0.6924 (SD 0.0432)
Global efficiency	0.7295	0.7341	0.7336 (SD 0.0520)
Small-world index	1.191	1.212	1.2634 (SD 0.2809)
Hierarchy	0.1369	0.1233	0.1271 (SD 0.0355)

## Discussion

The results in this unique case study show that the changes in SC following long-term DBS can be used together with advanced computational modeling to uncover the underlying neural mechanisms of DBS. Using the rare opportunity of having pre- and post-DBS DTI in a patient with PD, we investigated the functional consequences of changes in SC after long-term DBS. We mapped the optimum changes in connectivity weights from the implanted electrode in the STN and demonstrated that the optimal fit of connectivity weights from the STN to the putamen, caudate, STN itself and the thalamus was significantly changed the fit of the model, i.e., was significantly better than using no DBS. Importantly, we were also able to show that the functional changes induced by DBS not only led to clinical improvements but also significantly recovered the measures of segregation and integration towards normality. Specifically, our results show that DBS exerts its effects on network-wide brain functional connectivity through local Hebbian-like changes in specific connections from the STN.

We have previously shown significant structural changes following long-term DBS suggestive of a global change (van Hartevelt et al., 2014). The present results significantly extend these findings by showing that the STN. DBS is causing specific changes in the connectivity from the STN and that there is an optimum connection weighting of connections to the thalamus, caudate, putamen and STN. This is important as it offers novel evidence on the underlying neural mechanisms of DBS.

The present findings offer new insights into how DBS reaches its efficacy and could potentially be combined with the findings of a specific connectivity fingerprint for successful DBS cases (Fernandes et al., 2015). This might open up new possibility for future studies and might allow us to further elucidate the exact underlying mechanisms of DBS. Among other things, this might improve pre-surgical targeting as this would allow us determine which areas are important to be connected to the target for DBS in order to achieve a successful outcome.

Using the whole-brain computational modeling approach, as described in this paper, might also be applicable in other disorders. It could be a possibility to investigate how unbalanced functional connectivity networks might be restored, or rebalanced, to a healthy regime by altering specific weights or connections in order to find the optimal fit with a healthy functional connectome (Kringelbach et al., 2011). The potential benefits of this technique would not only be limited to neurodegenerative disorders but would be possible to extend to neuropsychiatric disorders (Deco and Kringelbach, 2014).

This is the first study investigating the underlying mechanisms of previously shown long-term structural changes (van Hartevelt et al., 2014) related to long-term DBS for PD. Although this data is unique and has allowed for the first time to investigate the underlying mechanisms of DBS *in vivo* using advanced whole-brain computational modeling, it should be emphasized that this is a case study. Due to the unique nature of DBS and the complications it brings forth, it is, at this point in time, extremely difficult to obtain more neuroimaging data for this particular patient group. Despite this being a case study, the results obtained from this data with the advanced graph analysis and whole-brain computational modeling has given us a unique insight into the potential underlying mechanisms of long-term DBS.

While the results of this unique case study using novel methods are exciting there is one important limitation linked to the imaging artifact introduced by DBS. The externalized lead on the left side of the brain causes a significant dropout in MRI signal leading to the limited use of only the right hemisphere. Although this is a limitation, it should be noted here that titration of the right electrode resulted in significant symptom improvement in the patient, whereas titration of the left electrode was more bothersome and was accompanied by adverse side effects. In addition, this case study also carries further potential limitations linked the course of medication typically found in advanced PD and especially changes in the medication regime during the long-term DBS period investigated.

It should also be noted that we did not specifically test different learning algorithms. Thus it is possible that the causal changes in STN connectivity could be achieved by other learning rules, which are not Hebbian-like. This would be of considerable interest to test in future studies.

Overall, this unique case study provides the first indication that DBS selectively changes the connectivity weights from the region where the electrode is implanted with consequences at the level of macroscopic functional networks. This is highly suggestive of neural Hebbian-like changes in white matter tracts induced by long-term DBS. This novel approach opens the possibility for computational models to predict the efficacy of individual DBS targeting pre-surgery and may even help identify novel DBS targets.

## Conflict of Interest Statement

The authors declare that the research was conducted in the absence of any commercial or financial relationships that could be construed as a potential conflict of interest.

## Footnotes

^1^http://www.fmrib.ox.ac.uk/fsl/
